# AI-driven decision support systems and epistemic reliance: a qualitative study on obstetricians’ and midwives’ perspectives on integrating AI-driven CTG into clinical decision making

**DOI:** 10.1186/s12910-023-00990-1

**Published:** 2024-01-06

**Authors:** Rachel Dlugatch, Antoniya Georgieva, Angeliki Kerasidou

**Affiliations:** 1 Ethox Centre, Nuffield Department of Population Health, University of Oxford, Old Road Campus, Headington, Oxford, OX3 7LF UK; 2 Usher Institute, Old Medical School, University of Edinburgh, Teviot Place, Edinburgh, EH8 9AG UK; 3 Nuffield Department of Women’s & Reproductive Health, University of Oxford, Level 3 Women’s Centre, John Radcliffe Hospital, Oxford, OX3 9DU UK

**Keywords:** AI-driven decision support systems, Artificial intelligence, Clinical decision making, Epistemic trust, Reliability, Epistemic authority, Cardiotocography, Qualitative

## Abstract

**Background:**

Given that AI-driven decision support systems (AI-DSS) are intended to assist in medical decision making, it is essential that clinicians are willing to incorporate AI-DSS into their practice. This study takes as a case study the use of AI-driven cardiotography (CTG), a type of AI-DSS, in the context of intrapartum care. Focusing on the perspectives of obstetricians and midwives regarding the ethical and trust-related issues of incorporating AI-driven tools in their practice, this paper explores the conditions that AI-driven CTG must fulfill for clinicians to feel justified in incorporating this assistive technology into their decision-making processes regarding interventions in labor.

**Methods:**

This study is based on semi-structured interviews conducted online with eight obstetricians and five midwives based in England. Participants were asked about their current decision-making processes about when to intervene in labor, how AI-driven CTG might enhance or disrupt this process, and what it would take for them to trust this kind of technology. Interviews were transcribed verbatim and analyzed with thematic analysis. NVivo software was used to organize thematic codes that recurred in interviews to identify the issues that mattered most to participants. Topics and themes that were repeated across interviews were identified to form the basis of the analysis and conclusions of this paper.

**Results:**

There were four major themes that emerged from our interviews with obstetricians and midwives regarding the conditions that AI-driven CTG must fulfill: (1) the importance of accurate and efficient risk assessments; (2) the capacity for personalization and individualized medicine; (3) the lack of significance regarding the type of institution that develops technology; and (4) the need for transparency in the development process.

**Conclusions:**

Accuracy, efficiency, personalization abilities, transparency, and clear evidence that it can improve outcomes are conditions that clinicians deem necessary for AI-DSS to meet in order to be considered reliable and therefore worthy of being incorporated into the decision-making process. Importantly, healthcare professionals considered themselves as the epistemic authorities in the clinical context and the bearers of responsibility for delivering appropriate care. Therefore, what mattered to them was being able to evaluate the reliability of AI-DSS on their own terms, and have confidence in implementing them in their practice.

**Supplementary Information:**

The online version contains supplementary material available at 10.1186/s12910-023-00990-1.

## Introduction

Recent advancements in artificial intelligence (AI) have the potential to drastically transform the medical landscape and improve the care clinicians can provide to their patients. Although medical AI is a conceptually broad category that encompasses different models (e.g. decision trees, deep learning, neural networks) and encapsulates various applications, from transcribing medical documents to diagnosing disease and even to performing surgery, one development of AI that is garnering more attention is clinical decision support. AI-driven decision support systems (AI-DSS) are intelligent computerized systems intended to help decision makers (e.g. clinicians) make more reliable decisions [1]. In a healthcare context, AI-DSS have the capacity to significantly enhance the information available to healthcare professionals, and in turn, improve the accuracy of disease diagnosis and other medical decisions [1]. These decision-support systems are not intended to act as autonomous decision-makers but as further sources of information from which clinicians can make informed, evidence-based decisions and recommendations.

Clinical decision making is a complex process. The cultural shift away from paternalistic paradigms towards shared decision making, in which patients make decisions jointly with healthcare professionals about their care, treatments, and tests, has added additional ethical, theoretical, and practical complexities to this process [2]. Although AI-DSS promise to improve the knowledge base from which clinicians form their decisions, they nevertheless introduce a new set of challenges. For example, some medical AI tools are already capable of outperforming humans in primary clinical tasks, as in the case of diagnosis of disease [3–5]. If AI continues to outperform humans, the epistemic authority of medical practitioners may be undermined, challenged, or supplanted altogether [6]. Moreover, if humans are no longer regarded as epistemic authorities, the role of healthcare professional also will likely change, becoming more about delivering empathetic and compassionate care than providing technical and medical expertise [7]. It has been argued that we have *already* reached a point where ‘anthropocentric epistemology is no longer appropriate because there now exist superior, non-human, epistemic authorities’ [8]. The proximity of this imagined eventuality is still up for debate, but the pace of AI development brings these questions to the fore.

Nevertheless, where AI-DSS are intended to be used as assistive tools rather than *substitutions* for clinicians and their expertise, healthcare professionals still retain the responsibility of synthesizing information, holistically assessing a patient’s clinical situation, and ultimately, making decisions collaboratively with their patients. A challenge for AI-DSS, then, is fulfilling the ‘conditions of trustworthiness’ (i.e., what it takes for clinicians to consider AI-DSS as trustworthy) so that they can be successfully integrated into decision making pathways [9]. More specifically, given that AI-DSS are meant to enhance healthcare professionals’ expertise, this challenge can be described within the framework of *epistemic trust*. Epistemic trust can be defined as ‘trust in communicated knowledge’ [10], and a ‘willingness to accept new information as trustworthy, generalizable, and relevant’ [11]. Fulfilling the conditions of epistemic trustworthiness is necessary for the successful implementation of AI-DSS, and ultimately for patients to reap their benefits. However, what constitutes these conditions is less evident.

This research empirically investigates the conditions of epistemic trustworthiness according to clinicians who may be tasked with integrating AI-DSS into their practice in the near future. This study is based on a speculative case study in the context of intrapartum care, a medical field where AI-DSS has the potential to improve healthcare professionals’ abilities to ascertain when it is appropriate to intervene in labor [12, 13]. Currently, medical practitioners primarily rely on cardiotocography (CTG), a tool that monitors uterine contractions and fetal heartbeat, to make risk assessments and decide whether interventions are recommended for the safety of the laboring person and/or baby (e.g. caesarean section, inducing labor, assistive tools) [14, 15]. CTG as an evaluation tool has significant limitations, but no better tools exist for continuous fetal monitoring in labor [16, 17]. Machine learning and AI approaches to CTG promise to improve the accuracy and reliability of these evaluations, and ultimately make risk assessing a more objective process. Leading the way in this field is the Oxford System (OxSys) [18], a decision-support system that combines clinical risk factors with CTG interpretation, to provide an objective risk assessment. Using data from 60,000 births and intelligent computer-based methods, OxSys holds the potential to provide cutting-edge clinical decision support. The aim of OxSys is to reduce the number of unnecessary interventions while also detecting risks and preventing serious perinatal outcomes. Moreover, it is hoped that if healthcare professionals can more reliably understand the risks to their patients with the help of OxSys, they could also facilitate better collaborative decision making.

In order for AI-DSS models like OxSys to improve clinical care on the ground, clinicians first must be willing to implement AI-DSS into their decision-making processes. For this reason, it is essential that clinicians’ perspectives are taken into consideration alongside the development and implementation of these systems. We have carried out this study with the aim of better understanding and amplifying their perspectives.

## Methods

### Aim of study

This study is part of a research project funded by the NIHR that aims to develop and validate a trustworthy and clinically reliable AI-driven CTG, the Oxford System (OxSys 3.0.)

Representing the ethics work package of this research project, this study investigates the perspectives of healthcare professionals who work in intrapartum care (i.e., obstetricians and midwives) regarding the introduction of AI-driven CTG on laboring patients. The purpose is to understand how healthcare professionals perceive this AI-driven CTG as (un)trustworthy, as well as the ethical issues raised by its introduction to in their decision-making processes, with the ultimate aim of developing an AI-driven CTG that healthcare professionals would consider trustworthy and reliable.

### Participants

Thirteen healthcare professionals took part in our study, of whom five were midwives and eight were obstetricians of varying seniority (including trainees, Junior Doctors, and consultants). Participants were recruited by cold contacting hospital trusts, distributing research flyers on social media, snowballing, and through known contacts in the NHS. Interviewees were based in Trusts throughout England. Interviewees have been given pseudonyms and their Trusts have been anonymized.

### Data collection

The primary form of data collection was semi-structured interviews, which allowed participants to identify the issues that mattered most to them. Interviews were conducted on Microsoft Teams, rather than face-to-face, as they took place during the Covid-19 pandemic. Online interviews enabled more healthcare professionals across the country to participate without being restricted by geography. Some interviews took place on interviewees’ days off work, but the majority of them happened in breaks during the workday. Participants were encouraged to read the Informed Consent Forms and Participant Information Sheets before interviews took place, and the researcher (RD) took informed consent verbally at the beginning of the interview, after reading through the form aloud. Interviews averaged 52 minutes in length and were voice recorded on an Olympus DS-9000, an external encrypted device.

Because OxSys was in development at the time of research, this study is based on a speculative design scenario. Participants did not use or engage directly with OxSys or another AI-driven CTG as part of this study, but instead reflected upon how using a tool like OxSys might impact their clinical decision making, and what it would take for them to actively want to use such a tool in their care. Interviewees were first prompted to describe their main professional responsibilities, their relationships with patients and other healthcare professionals, and their use of CTG. They were then encouraged to reflect upon their decision-making processes with regards to interventions in labor and the role of CTG within that process. After being asked what could be improved upon with the types of CTG they have used and what would make it more trustworthy, participants were given a brief description of an AI-driven CTG, OxSys. The interviewer followed up with a series of questions about how this AI-driven CTG might change how they care for patients or make decisions about interventions, what they would need to know about it before determining its trustworthiness, and if they had any concerns about introducing this kind of AI into their decision-making processes. Discussions pertaining to themes like trust, trustworthiness, and reliability were given most attention in these interviews. A sample topic guide used for these interviews can be found in our supplementary materials, but it should be noted that the interviewer asked follow-up questions based on each interviewee’s answers; as such, no one guide can cover all questions asked.

We conducted interviews until we reached data saturation, the point at which collecting more data no longer generates new insights or information [19]. While quantitative studies rely on greater samples, in qualitative studies, when participant groups or objectives are narrowly defined, data saturation can be reached between nine and 17 interviews [20]. Our study, where data saturation was reached in 13 in-depth interviews, falls into the middle of this window. In addition, our approach to using semi-structured interviews afforded the researcher flexibility to explore unforeseen topics and interests raised by participants, personalize the conversation, and establish rapport in ways not possible in more rigidly-structured, quantitative studies [21].

### Analysis

Interviews were transcribed verbatim. With the use of NVivo, a data software for qualitative research, transcripts were coded and analyzed with thematic analysis. Thematic analysis is a method for interpreting qualitative data that draws meaning from recurring patterns and themes in the data [22].

## Results

### Accurate and efficient risk assessments: preventing adverse labor outcomes and having confidence making decisions

It emerged from interviews that the most important capability for an AI-driven CTG, according to our participants, was accuracy and efficiency in making risk assessments. As participants shared, obstetrics is a high-stakes profession. These healthcare professionals are responsible for not only one life but two (or more). The high-risk, high-stakes environment in which they operate is also reflected in the fact that obstetrics is responsible for a considerable portion of all litigation costs in the UK [23]. Therefore, being able to accurately predict when an intervention is needed in a timely matter is of utmost importance to save lives, prevent unnecessary morbidities, and also avoid costly legal battles.

One interviewee shared that although public perception is that childbirth is generally safe, giving birth is still risky:

So, I think if you go back a hundred years, it wasn’t unusual to die in labor and childbirth and pregnancy. And fifty years ago, I think people still remembered that and therefore, medicine was much more patriarchal, and we told people what to do, that wasn’t right. But I think in the last twenty, thirty years, there’s been a real shift towards birth being an experience. And that at times that can cause conflict because it’s never viewed as a high-risk thing to do in society. But there are still lots of risks of having a baby. And with an increasingly co-morbid and medically complicated society and kind of as a whole with getting more unhealthy, the expectations are still that you’ll have a completely low-risk labor and birth. Marrying those things up can be really difficult. (Obstetrician1)

Another obstetrician reiterated Obstetrician1’s point that giving birth is still risky, and because of this risk, their main responsibility is to assess that risk and set them on the appropriate pathway (e.g., high- or low-risk pathway). Several participants also emphasized that not only is this risk paradigm essential for how they make decisions, but risk *aversion* is a driving force behind the profession. Obstetrician2 said that obstetrics is ‘probably the single most risk averse specialty,’ and that in her practice, ‘I don’t want low risk, I want none.’ Although participants spoke of keeping their patients safe and healthy as a major priority, they also shared that ‘fear of litigation’ contributes to this risk aversion.

Given how vital risk assessments are for healthcare professionals in determining whether interventions need to take place to prevent adverse labor outcomes (and avoid costly legal battles), it is understandable that when confronted with the possibility of using AI-driven CTG, our participants focused on whether the system could improve upon the accuracy and efficiency of risk assessments. Being able to identify risks—and to do so quickly—could enable healthcare professionals to take appropriate actions swiftly, preventing unnecessary injuries or mortalities. Consider the following comments about what would make an AI-driven CTG worth using:

… but [if] we’re improving the way in which we communicate with [patients] and we’re able to *give more accurate risks*, I think that would be really cool. (Obstetrician3)

Yeah, I mean, if you could get research that showed that, we were going to be safely monitoring babies *and it would actually, accurately predict a baby that’s going to need support.* Maybe even before the mums in labor, you know? [...] Rather than you having to be in labor for 24 hours and, you know, having all of the issues associated with that before we go for section. (Midwife1).

Like *something that wasn’t previously picked up on, being picked up on*. Which then resulted in a healthy, a healthy delivery. (Midwife2).

Well, we, I mean, [if] *we could pick up on things earlier*. […] In terms of, ‘cause some interventions are necessary, people, you know, people give interventions a bad rep, but in certain situations if babies aren’t born very quickly they will die*.* (Midwife5).

One obstetrician even said, ‘It’s all about accuracy,’ when asked what was most important to him, noting that accuracy enables him to have confidence and certainty in the decisions he makes. Obstetrician4 also said that it would ‘give you some confidence’ to make decisions assisted by AI that has been proven to be more accurate than humans. As Obstetrician3 said, more accurate risk assessments go hand-in-hand with improved communication and collaborative decision making, too. Nevertheless, many participants said that even though AI-driven CTG might make them feel more confident in the accuracy and efficiency of the risk assessments, they still believed that the final responsibility—and the accountability that goes along with those decisions—should belong to them.

I mean, [no matter] how much of a technology … we bring into the service*, ultimately it’s our brain that thinks what is right for the patient. And we have to take the responsibility*. (Obstetrician5)

But equally *I don’t think you can 100% rely on it,* you have to be still, you know, it’s still, let’s say *if you had to go to court you can’t say, ‘Oh the computer told me it’s okay.’* (Obstetrician4)

Despite believing that the responsibility should remain with healthcare professionals, participants were excited by the prospect of AI-driven CTG that could improve their ability to identify risks to their patients and act accordingly on this information.

### Personalization and individualized medicine: recognizing differences

Because patients are diverse, the tools that healthcare professionals use to care for their patients, should be able to reflect this diversity in clinical needs. For this reason, many participants said that they would want the ability to input clinical characteristics for their patients into this AI-driven CTG, and that they would prefer a composite score risk that accounted for all of these differences over a report that considered the fetal heart rate alone. This personalized AI-DSS would enable medical professionals to share more individualized risk assessments with their patients. Nevertheless, participants also acknowledged that even though trustworthy AI-driven CTG should be personalizable, personalization does not begin and end with technology and AI-driven tools.

When asked about where obstetrics should be heading, almost every participant mentioned the importance of personalized and individualized medicine. As Obstetrician5 said, ‘it’s not one-size-fits-all.’ Moreover, several participants discussed personalized medicine in the context of AI-driven CTG, and noted that they would want a machine which enabled them to input different clinical factors (based on the patient and their needs), as well as develop a holistic assessment that considers the whole picture of the laboring person in front of them.

[This is where] the personalized care comes [in]. Your algorithm, what you are creating, if that takes into the whole clinical picture into consideration [.] I think most important, as a, obstetrician being in this field, for me I would like to see the whole clinical picture, not [only] the CTG. (Obstetrician5)

Obstetrician3 expanded upon this idea and spoke about personalizing risk assessments by integrating all clinical factors into a single risk score, rather than looking at the fetal heart rate separately from the other relevant clinical factors, and then determining risk after the fact.

… at the moment what happens is we, we interpret the CTG and then we look at the other features, rather than integrating her risk factors and other temperature, progress, meconium-stained like or with the fetal heart rate features. And then coming up with a composite view of where she is. So … that [composite view] makes much more sense rather than doing one and then the other. (Obstetrician3)

What Obstetrician5 and Obstetrician3 are talking about, ultimately, is the ability to individualize risk assessments. While healthcare professionals are already attempting to individualize their risk assessments (given that currently they integrate both fetal heart rate measures, as well as other clinical factors, into their assessments), a technology that would consolidate this process would enable them to more efficiently do what they currently aim to do: tailor assessments to the individuals in front of them.

However, it is worth acknowledging that while healthcare professionals want an AI-driven CTG to be personalizable, they nevertheless did not see personalization beginning and ending with technology and AI tools. One midwife (Midwife1) emphasized the importance of building relationships with their patients, and how personalizing their care has an element of ‘artistry.’ Building this kind of interpersonal relationship cannot be achieved by technology alone, because personalization requires getting to know a person, their values, preferences, and personality.

So, I really strongly feel, and the research supports it, it’s what I’m doing my PhD on, is continuity of midwifery care, relationship-based care, getting to know one person or, you know, maybe two midwives. We know that it improves people’s outcomes, and we don’t really know why. And, like, technology can’t improve that. They can’t improve you feeling connected to someone. They can’t improve you just noticing something slightly different about a person. And maybe acting on your instinct, which isn’t quantifiable at all. (Midwife1)

Regardless, it is evident that the healthcare professionals we interviewed were united in wanting to pursue more personalized-based medicine, and that individualizing their care was becoming a growing priority in their practice. And although making decisions also requires getting to know people and ‘acting on your instinct,’ as Midwife1 said, a personalizable AI-driven CTG was perceived as beneficial to their practice.

### Outcomes over institutions: the importance of reliable and improved clinical outcomes

Although some studies have shown that the general public favors AI developed in the public sector [24, 25], the healthcare professionals we interviewed were broadly indifferent to the type of institution responsible for developing the AI-driven CTG (i.e., public sector organizations versus private companies). Instead, interviewees emphasized the importance of the AI improving outcomes for their patients while downplaying the relevance of who had developed the technology. Nevertheless, this is not to say that which institution had developed the tool was perceived as altogether inconsequential. Several interviewees said it might be able to get patients on board with AI if it had been developed in the public sector or by a university, rather than a private company, due to public distrust of for-profit corporations. However, this was raised more as a pragmatic concern, rather than something to which healthcare professionals in our study gave significance themselves.

Consider some of the following responses when asked directly about whether it would matter who had developed the tool:

No, probably not. As long as it’s, you know, *as long as it works and it’s reliable…* I don’t think I would judge on, you know, who developed it, no. (Midwife3)

Good question. Not, not, not massively to me. As long as it, *as long as it worked.* (Obstetrician3)

This idea of ‘as long as it worked’ was echoed amongst healthcare professionals, underlining the importance of the reliability of the system over the type of institutional association. Several interviewees elucidated further what it means for the product to work successfully and reliably. The following quotes illustrate this point:

…if it had been *recognized as, you know, helpful in high-risk populations,* then yeah, more power. Gosh, I mean it would be great. (Obstetrician6)

But then it’s got to be that it actually works, you know, in a pragmatic sense. When you put, when you, you know, use them in a clinical setting. And I think there’s one thing developing technology, there’s another in, in ensuring that when you put it in a clinical setting, it, it remains stable as an intervention. And equally, it’s easy to use, it’s well received, you know, it, and, and we can demonstrate some improvement in actual clinical outcomes. (Obstetrician3)

Our interviewees had mixed responses about whether they thought private or public companies would be most able to deliver a reliable product that improved patient outcomes in clinical settings. Some participants believed that because a private company would have more funding than an underfunded university, they might have more capacity to develop smarter technology. Others said that they trust universities, like the University of Oxford, because of its reputation as a renowned research institution and affiliation with Professors Dawes and Redman, creators of the Dawes-Redman, a computerized CTG. One interviewee said that private companies and universities were likely to have the same type of researchers, who would also be ‘experts in that field,’ so there was no reason to think one type of institution would be more capable of developing reliable AI than the other. Interestingly, despite the fact that participants did not agree on which institution would create a better product, the discussions about whether they would prefer one type of institution to develop the product over another always came down to the same thing: a debate over reliability and delivering greatest improvement in clinical outcomes.

Still, a handful of interviewees mentioned that the type of developer might matter to their patients, who could be wary of technology that had been made for the purpose of profit over public good. One participant added that it would be even easier to ‘sell to patients’ if the technology had been researched and developed at a British university, because she could tell them ‘it was developed on [and] with women like them.’ However, these conversations had more to do with how they could reassure their patients with AI rather than whether than they themselves would deem it trustworthy.

### Transparency in the development process: ethical frameworks and rigorous research practices

Nevertheless, just because our participants downplayed the significance of the developer does not mean that they were not interested in how the tool itself had been developed. On the contrary, they expressed a considerable amount of interest in *how* the research had been conducted and *how* the product had been developed. Being subject to the proper ethical frameworks and high research standards were essential to how reliable and trustworthy they perceived the AI in question. Additionally, it can be inferred from participants’ comments that it was not sufficient to be told that AI was reliable, accurate, and efficient; they wanted to be able to verify for themselves that the research had been conducted to the highest possible standards. As such, transparency—in the research, development, and validation processes—was important for healthcare professionals to have confidence in the reliability of the AI. Moreover, they felt that this transparency would enable them to better understand the tool’s capabilities and limitations. In practice, this would mean that they would know in which circumstances it would most benefits their patients (and when or for whom it was not suitable), thus giving them more confidence in the usage of the tool within their own practice.

The following quotes highlight that despite not being concerned about *who* had developed the AI, our participants were very concerned about *how* it was developed, seeking transparency in the development process—from ethics to the nitty gritty of the research itself:

It’s more around, *not who, it’s the what process, what ethics, what cohort, what population size, how is it conducted*? [….] so, it’s more around the how transferable is it? How robust is it basically? The why, it’s the how did you get the results that you’ve got and how valid are they? (Midwife4)

I suppose the, the percentages, kind of, *sensitivity and specificity for picking up a fetal hypoxia, how many CTGs it has looked at, what would have been the margins of error as well*. Yeah, and I think it’s, it’s, like, in some kind of clinical cases where maybe the management clinically was difficult and the CTG helped, *just to help me understand the, kind of, day to day kind of role in labor.* (Obstetrician4)

Interestingly, as these quotes illustrate, our participants were not only concerned with research results but *how* the research team got to those results. This is because they want to evaluate, verify, and or/challenge the results of the study and make up their own mind about its clinical applicability. Midwife4 added that it was also important to understand the research trials, how they had been conducted, and on which populations they had been trialed so that they could understand ‘under what circumstances does it work’ and if there are ‘circumstances where it doesn’t work.’ While almost every participant spoke about the importance of using only evidenced-based medicine, what was revealed in these interviews was that they wanted to review the evidence and deliberate for themselves. Are the research results valid, and in what contexts are they valid? As such, the importance of transparency cannot be understated for healthcare professionals. Understanding the research and validation process is what enables them to have confidence in the technology and consider it reliable. Moreover, transparency in these processes reveals to healthcare professionals where and how it is appropriate to integrate the system into their clinical decision making because they can compare the research context(s) to their own clinical setting and patients.

Finally, several healthcare professionals spoke about the importance of regulatory ethical frameworks for the research and trial phases of the product development. They spoke of the importance of having ‘ethics approval,’ ‘that it had been done in a very ethical way,’ and that it had been subject to ‘the full regulatory research ethical framework.’ While our participants went into more depth about transparency in research design and trial validation, they nevertheless wanted to know that research had been conducted in an ethical way, something they felt would be satisfied by adhering to codes of conduct and ethics governance.

## Discussion

Healthcare professionals need to know that any new technology they integrate into their practice will help them deliver the best possible care to their patients. What emerged from our interviews was the importance of AI systems and tools being reliable. Our participants’ desire for reliability was expressed in their comments about wanting accurate, efficient, and personalizable risk assessments, in their emphasis on the tool’s proven clinical results over any moral judgment of its developers, and in their need for transparency and rigor in the tool’s development and clinical validation. Particularly, the healthcare professionals interviewed for this study were concerned about being able to rely on AI tools for correct and accurate information about their patients, information that they will be able to use in their assessment of whether and when to intervene during labor.

We can call this attitude epistemic reliance, when A accepts a proposition x to be true or correct on the basis of B’s testimony. Epistemic reliance, as understood here, however, is distinguished from epistemic trust on social rather than epistemological (i.e. propositional or doxastic) grounds (for a comprehensive account of the latter see: Goldberg, S.C. (2010) [26]. In this sense, although epistemic trust also necessitates some form of reliance, epistemic trust is more than merely depending on someone in order to form one’s beliefs [27]. As McCraw (2015) explains when we epistemically trust someone in the form of A epistemically trusts B for A’s belief x, A accepts x to be true *because of* B, meaning that certain affective and normative expectations are ascribed to the trustee [27]. However, it was clear from our interviews that clinicians and other healthcare professionals were not seeking to *trust in* the AI-DSS system but wanted to know that they could rely on it or, to use McCraw’s term, *trust that* AI-DSS proposition was true.

Additionally, the healthcare professionals’ interviews here ascribed no affective or normative expectations to the tools or the institutions who designed them. Studies have shown that patients and the public often express certain expectations towards institutions and companies who develop medical AI tools, particularly that these institutions and companies serve the common good [24, 28]. By contrast, healthcare professionals in this study explicitly stated that neither the nature (public or private) nor their intentions (for profit or not-for-profit) mattered as long as the tools worked. This further supports the claim that what these healthcare professionals are seeking from these tools is not epistemic trustworthiness but rather epistemic reliability. They are not prepared to *trust in* these tools but rather *trust that* these tools work.

Furthermore, the epistemic reliability of the AI model was not something that our interviewees were prepared to accept uncritically. Determining the reliability of AI-DSS was something that clinicians believed to be part of their duty to deliver safe and appropriate care. This point was seen in the fact that healthcare professionals consider as part of their role to understand the AI model’s limitations and capabilities, as well as to make judgments about the applicability of the AI-DSS to the particular patient in front of them. This means that it is not the reliability of the technology alone that matters, but the clinicians’ *confidence* in its reliability, something determined by clinicians’ own methods of questioning, understanding, and interrogation of the AI-DSS and its validation process. The importance of clinicians making their own conclusions about reliability underscores the fact that, at the moment, no reliable and widely accepted global processes and systems exist to validate medical AI [29, 30]. Until such processes are developed and themselves validated, it is likely that healthcare professionals will remain, justifiably, critical towards new AI tools that are introduced into their healthcare space.

Despite questions raised in AI literature about the potential diminishing role of healthcare professionals alongside AI [31–33], our study suggests that even in an AI-assisted healthcare context, their epistemic authority remains essential for administering patient care. Healthcare professionals’ epistemic authority is based on the fact that they have the requisite skills, ability and know-how, and also access to the evidence and resources required for them to provide appropriate and safe care to their patients [34]. As such, determining when, where, and how it is clinically appropriate to apply AI-DSS is still a matter of exerting epistemic authority, one that clinicians perceive as being within their remit. The fact that our interviewees did not raise concerns about being replaced by AI, as well as the assertion that they should still have responsibility (including legal responsibility) for their patients, reiterates the point that even with the use of AI-DSS, clinicians still perceive their epistemic authority as relevant to the decisions being made with their patients. This authority underlines clinicians’ sense of duty to their patients, which was reiterated time and time again in our interviews (recall Obstetrician 5, who said, ‘*…ultimately it’s our brain that thinks what is right for the patient. And we have to take the responsibility*.) This might go some way to dispel emerging concerns about AI deference, and a new age of AI paternalism that have been expressed in the literature [35, 36]. Of course, this remains an empirical question, and it is still unclear whether healthcare professionals will sustain this attitude of epistemic authority and superiority over AI tools once these tools are in place.

That clinicians retain their sense of epistemic authority has further implications for the integration of AI-DSS into healthcare. Although the bar is arguably high for when clinicians would want to incorporate AI-DSS, it is not the case that AI needs to be fool proof, either. Understanding the limitations of the AI *is* essential for clinicians to feel confident in using these new systems. One could argue that it is as important for clinicians to understand the limitations and capabilities of AI-DSS as it is for the technology in and of itself to be improved upon.

Finally, the fact that the healthcare professionals are not considering AI-DSS tools as epistemic authorities lends further support to the argument that what they are seeking from AI is not epistemic trustworthiness but rather reliability. If clinicians would consider AI as being the epistemic authorities is their context, then they would need to be prepared to change their beliefs based on the AI’s proposition [37]. However, what these healthcare professionals are saying is that proviso that the AI-DSS is reliable, they would incorporate it into their decision-making process. Thus, they would retain epistemic authority in the healthcare context.

### Implications for OxSys development

At the time of this study, OxSys was still in development. This means that our findings could be funneled back to the OxSys developers, and that elements of the tool and the plan for its implementation could be adapted in accordance with our findings. A workshop was held with the ethics team, development team, and public and patient involvement team to brainstorm ways in which our results could be incorporated into OxSys’ development so that healthcare professionals would feel confident implementing it into their daily clinical practice. Some of the topics discussed included: how the research team can best demonstrate that OxSys is more accurate than current clinical practice, and how should this information be communicated to clinicians; adding a functionality in the model that prompts questions to healthcare professionals to consider various risk factors; whether OxSys should be described as a ‘decision-support’ tool instead of ‘AI,’ the latter of which might invoke incorrect imaginaries of autonomous agents; establishing a 24/7 call center to support on the ground use of OxSys; and budgeting costs for training HCPs in the use of OxSys.

One of the main benefits of this study is that it enabled OxSys developers to understand some of the most important factors for creating a trustworthy AI-DSS from the perspective of clinicians before even being launched. While more considerations will likely arise if/when OxSys is used in clinical practice, our speculative study enabled the development team to get a head start on addressing clinicians’ concerns and factoring in the values of prospective end users.

### Limitations

Due to limitations in sample size as well as restrictions on collecting personal data, we were not able to take into consideration how working in different NHS trusts impacted clinicians’ perspectives on AI-DSS. Further research could expand upon this study by exploring how the hospitals where people work—including the technology available them in these places, their ratio of staff to patients, and more—affects their perspectives on AI and decision making. Moreover, although our study touched upon some differences between perspectives midwives and obstetricians, as well as differences within these groups based on seniority, further research could draw more attention to these divergences and their implications for integrating AI-DSS.

In addition, this research did not consider perspectives from clinicians currently using AI-DSS in the context of intrapartum care. This study was based on a speculative design scenario, because OxSys was not being trialed in clinical settings at the time interviews were conducted. Our study has value in that it allows clinicians’ perspectives to be considered before clinical trials take place; nevertheless, sustaining this kind of research is important throughout the validation process. It would be worthwhile to conduct further research that observes how and if clinicians’ perspectives change when using this kind of technology.

Finally, given that our participants wanted to verify the reliability of the AI system themselves, questions arise as to how non-AI experts can best evaluate the accuracy of these systems. Future empirical research could explore how clinicians make judgments about the accuracy of new AI systems in practice (and how accurate clinicians are in making these judgments). More theoretical research could unpack the tension between human and machine skills, including the potentially fraught undertaking of humans assessing machines and systems with capabilities superior to humans.

## Conclusion

In this research, we explored clinicians’ perspectives on the trustworthiness of AI-DSS. More specifically, this research investigated the conditions that need to be met for obstetricians and midwives to want to introduce AI-driven CTG into their decision-making processes around interventions in labor. We concluded that instead of trustworthiness, what the healthcare professionals interviewed seek from the AI tools were reliability. Several factors were echoed in clinician interviews as being essential for AI-DSS being considered reliable: producing accurate and efficient risk assessments (so as to prevent adverse outcomes), having personalization capacities (so differences between patients can be incorporated into decision making), proof of improved clinical outcomes (so clinicians have confidence it works on the ground in real life scenarios), and transparency in the development process (to ensure that the research has been rigorous and done according to ethical frameworks).

Nevertheless, even where AI is used to assist healthcare professionals in clinical decision making, it remains the fact that people, not machines, are tasked with delivering care. Therefore, even though there are conditions of reliability that AI-DSS must fulfill for healthcare professionals to integrate them into decision-making pathways, what matters even more is that there are people who can evaluate the reliability of these systems and have the expertise to implement the systems appropriately. It is for this reason that this study is important—not because it delineates a set of expectations that AI-DSS should per se, but because it highlights that clinicians need be able to evaluate the reliability of AI-DSS on their own terms before integrating them into practice. Only when clinicians feel confident that they can rely on AI-DSS can patients reap their benefits, which is the ultimate goal of introducing these technological advancements.

### Supplementary Information

**Additional file 1.**

## Data Availability

Data relevant to the research are included within the manuscript. As per MS IDREC requirements, data from the study will be destroyed 3 years after the end of the study. Because participants did not consent to data archiving, the data used for his study are not public available. However, specific data can be made available upon request from the first author.

## Abbreviations

AI: Artificial intelligence
AI-DSS: Artificial intelligence decision support systems
CTG: Cardiotocography
