# Supplementary material for: AI-driven decision support systems and epistemic reliance: a qualitative study on obstetricians’ and midwives’ perspectives on integrating AI-driven CTG into clinical decision making
Source: BMC Med Ethics. 2024 Jan 6;25:6. doi: 10.1186/s12910-023-00990-1 (PMC10771643; doi:10.1186/s12910-023-00990-1)
Supplement: Supplementary file 1 — Additional file 1. [file 12910_2023_990_MOESM1_ESM.docx]

***INTERVIEW TOPIC GUIDE FOR HEALTHCARE PROFESSIONALS***

***Notes***

*This is a topic guide. Specific wording of questions will be adapted as the project progresses and according to the participants’ answers. This topic guide will be used as a map for the researchers to ensure that they cover all important topics, even if they will have to adapt either the order of the questions or the way they ask the question.*

**Introduction**

- Researcher/s introduce the aim of the research
- Researcher asks if participant has any further questions regarding the project and their participation
- Researcher confirms consent
- Research reminds participant that they are free to refuse to answer any questions, or to stop the interview at any stage
- Researcher thanks participant for taking time to do this interview

**Introductory questions**

What is your role (title) and training?

How long have you been working as a [Obs & Gyn Consultant/Midwife/Specialist Nurse]?

Where are you based/which trust?

What are the main parts of your job and your key responsibilities? How would you describe your job? What made you want to go into this field?

**Decision-making during labor and delivery**

What do you see as your main responsibilities during labor & childbirth? How are those responsibilities different from the other healthcare professionals in your team? (Are there misconceptions about your responsibilities/your job?)

How does it feel to have those responsibilities?

How would you describe your relationship to someone who is pregnant and/or in labor when you’re providing their care? How do you develop that relationship/how does it change over time?

How much choice and of a say should the pregnant person have in decision making about their pregnancy and delivery?

What are beliefs (or philosophy) about about birth?

Can you describe the most ideal labor and delivery? What would that look like from your perspective?

What factors might stop that from happening in real life?

How do you feel about home/water births v hospital?

How do you know when something is wrong?/or do you get a hunch something isn’t right?

How are decisions made about whether intervention is necessary when someone is in labor? What is your role when decisions are being made about whether to intervene? How does it feel when those decisions are being made? (How confident are you in making those decisions?)

From a diagnostic point of view, how easy are these decisions to make? Do you feel like they’re usually accurate?

How does it feel to make those decisions? How collaborative are they? How does it feel to tell someone in labor that a decision needs to be made/communicate these decisions? How well are those decisions communicated?

How often do you need to make this type of decision?

What decisions that are most challenging to make during labor? Is there an example you could give me?

What are the most straightforward decisions to make?

What would make it easier to make decisions during labor?

What are your thoughts about electronic monitoring like CTG, and when should it be used?

What do you take into consideration about whether you need to intervene? What role does CTG play? How is this communicated/discussed with the patient?

What would make CTG better to use?

What experiences do you have with the Dawes-Redman, if any?

Why do you think mistakes are made about intervening—like intervening when it wasn’t necessary? What are the diagnostic challenges?

To what extent does litigation impact decisions around intervention?

**Emotional impact of work**

How do you look after yourself when it’s been a challenging day? What kind of support do you receive?

Does lack of support/working too many hours affect how you can do your job? How does it affect you?

How do you feel going into another delivery after a difficult one?

What other kind of information or support would allow you to do your job better?

What’s the most difficult aspect about your job?

What’s most rewarding about your job?

What kind of changes would you like to see in intrapartum care moving forward?

What do you wish people know about your job?/OR anything related to your work?

A lot of women & people who have been through childbirth have said they feel like they weren’t listened to. Why do you think this is?

How has Covid had an impact on your job?

**Artificial intelligence**

*[Transition: As you probably know, there are more and more AI tools developed to be used in healthcare, including in prenatal care.]*

What comes in mind when you hear the term ‘artificial intelligence’?

What do you know about artificial intelligence? (probe: how it works, where you find it, what it is used for?)

How do you feel, generally about the use of artificial intelligence in healthcare? Why?

How do you feel about the use of artificial intelligence in perinatal care? Why?

Have you encountered any kinds of artificial intelligence in healthcare previously? How was that experience?

**OxSys**

[Researcher briefly describes OxSys; explains *the type of AI tool I have in mind here is one that uses data from many patients to provide more accurate predictions about when to intervene in labor*]

How would you feel about using such an AI tool in your practice? Why? [probe: confident, able to rely, happy to use the guidance, just another point of information to take into account?]

What would you want to know before using it?

How do you think it would change your experience of providing care during labor and delivery?

How do you think it might impact on your relationship with your patient? Why?

How might it impact the accuracy of diagnosis?

What do you think are the advantages of using this kind of AI tool when you are in the delivery room? [probe: as an example; what do you think about the increased accuracy of prediction? Is this important? Valuable? Any other things that can be important and/or valuable]

What kind of issues or challenges do you anticipate?

Thinking back to earlier in our discussion, do you think it would bring you closer to your ideal labor and delivery scenario?

Do you have any thoughts on algorithmic bias or how reliable the tool would be for all types of patients?

Which patients might be more/less comfortable with it?

What type of patient information would you want to input into the tool?

Does knowing who is developing this tool, e.g. if it is university researchers or an international private company like Google or IBM, make any difference to how you might think about it? Why?

Would you feel comfortable overriding a decision?

What might be the impact on your workload?

What kind of training or protocols would you want in place?

**Ending:**

Is there anything you’d like to talk about that I haven’t asked you about yet?

I will be interviewing people about their experiences of giving birth and their feelings about artificial intelligence, too. Is there anything you think I should ask them?

Would it be okay if I share your email address with our finance team so that we can send you a voucher for your time?

Anyone else who wants to participate?

Any final q’s?/ thank participant for their time
